# Long COVID symptoms in the COVID unit of the emergency Department of Abderrahmen Mami Hospital in Tunisia: prevalence, main symptoms, and associated factors

**DOI:** 10.11604/pamj.2025.50.109.37221

**Published:** 2025-04-23

**Authors:** Nawel Dhaouadi, Donia Souissi, Sarra Ben Yacoub, Afef Skhiri, Chahida Harizi, Radhouane Fakhfakh, Rafik Boujdaria

**Affiliations:** 1 Department of Epidemiology and Statistics of Abderrahmen Mami Hospital, Ariana, Tunisia,; 2 Department of Emergency of Abderrahmen Mami Hospital, Ariana, Tunisia,; 3 Faculty of Medicine of Tunis, University of Tunis El Manar, Tunis, Tunisia

**Keywords:** COVID-19, post-acute COVID-19 syndrome, prevalence, cohort study, risk factors, Tunisia

## Abstract

**Introduction:**

the COVID-19 pandemic has been evolving since 2019, affecting over 536 million individuals and causing more than six million deaths. After the acute phase, the onset or persistence of symptoms grouped under the name of long COVID is reported. The variability of symptomatology makes this a relevant subject of study, since more than one million cases of COVID-19 have been reported in Tunisia. The aim of our study was to determine the prevalence and potential risk factors for long COVID.

**Methods:**

we conducted a retrospective cohort study of patients admitted to the COVID unit of the emergency department of Abderrahmane Mami Hospital in Tunisia from April 1st to August 1st, 2021. The National Institute of Health Care definition of long COVID (November 2021) was adopted.

**Results:**

overall, 1,271 patients were admitted during the study period. After excluding deceased and unreachable patients by telephone, 454 were included in the analysis. The mean age was 58.6 ± 13.9 years, with a male predominance (53.7%). The prevalence of long COVID was 84.8%. The most common manifestations were breathing discomfort, asthenia, memory problems, tiredness, and arthralgia. In multivariable analysis, female sex was identified as a risk factor for long COVID (aOR: 1.732, 95% CI 1.002-2.995; p = 0.049).

**Conclusion:**

the high prevalence of long COVID observed in our study highlights the need for post-COVID follow-up among affected patients. Our findings suggest that women had a higher risk of developing long COVID, indicating the benefit of individualized monitoring and care programs for this group.

## Introduction

The severe acute respiratory syndrome coronavirus 2 (SARS-CoV-2) represents a fundamental concern for the healthcare field. The first case was detected in December 2019 in China [1]. The World Health Organization (WHO) officially declared COVID-19 a pandemic in March 2020. Until April 2022, there have been over 498,235,183 million reported cases of COVID-19 worldwide, with several deaths surpassing 6,175,800 [2]. The clinical presentation of SARS-CoV-2 was very heterogeneous; it varies from asymptomatic to severe forms. Various studies described the acute presentation of a COVID-19-infected patient well [3]. The majority of patients presented with fever, sore throat, cough, shortness of breath, and chest pain [4]. The COVID-19 symptoms can affect any body system, including the respiratory system, the brain, the cardiovascular system, the liver, the kidneys, and the gastrointestinal system.

Strategies such as social distancing, individual protection for the population and workers, assistance flows, intensive care beds, specialized human resources, medical equipment, and vaccination remain a growing challenge, as the epidemic progresses in waves, with different intensity and temporal distribution in different countries [5]. The pandemic was able to overcome these preventive barriers and continue to spread, leaving behind acute but also long-term consequences. Indeed, a significant number of patients who have recovered from COVID-19 have reported lingering symptoms affecting their daily lives, including fatigue, shortness of breath, joint pains, memory impairment, and insomnia [6].

There is a misconception that these symptoms remain only for patients with severe clinical forms, whereas these symptoms also remain for asymptomatic patients. Since the etiopathogenesis remains unclear, scientific research is in the process of elucidating the spectrum of long COVID (LC). There is no clear definition of this syndrome. Several definitions have been formulated. The National Institute for Health and Care Excellence (NICE) defined it as a constellation of « signs and symptoms that develop during or after an infection consistent with COVID-19, continue for more than 12 weeks and are not explained by an alternative diagnosis » [7]. However, the WHO defined LC as « a condition that occurs in individuals with a history of probable or confirmed SARS-CoV-2 infection, usually three months from the onset of COVID-19 with symptoms, and that lasts for at least two months and cannot be explained by an alternative diagnosis » [2]. It would be very interesting and enriching to describe this syndrome in the African population, especially the North African one, for which there are, until now, no published data.

Tunisia represents one of the most affected countries by this pandemic and is ranked 59^th^ on the world scale of the most damaged countries. Until April 2022, there have been over 1,037,358 reported cases of COVID-19, with several deaths surpassing 28,425, and 530,545 recovered cases [2]. This study aimed to determine the prevalence and risk factors of LC among hospitalized patients affected by COVID-19 in the COVID-19 unit of the emergency department of Abderrahmen Mami Hospital in Tunisia.

## Methods

**Study design and setting:** a single-center retrospective cohort study with a three-month follow-up period was conducted in the COVID unit of the Abderrahmen Mami Hospital emergency department between April 1^st^ and August 1^st^, 2021. The Abderrahmen Mami Hospital, located in Ariana, Tunisia, was established in 1992 with a hospitalization capacity of 374 beds. It is a reference center for respiratory diseases in Tunisia, and plays a crucial role in the management of respiratory infections, chronic lung diseases, and, more recently, COVID-19, serving as a referral center for patients from all over the country.

**Study population:** our study included all adult patients diagnosed with COVID-19 and admitted to the COVID unit of the Abderrahmen Mami Hospital emergency department between April 1^st^ and August 1^st^, 2021. COVID-19 was confirmed using either real-time polymerase chain reaction (RT-PCR), rapid diagnostic test, or chest tomography scan. Only patients with a minimum period of three months between admission and the start of the study were considered. We excluded patients who died during or after hospitalization, those without available phone numbers, those who did not respond after three call attempts, and those who declined participation. All eligible patients meeting these criteria were contacted.

**Data collection:** in this retrospective cohort study, data were collected in two stages-during the acute and post-acute phases of COVID-19-using a structured approach to ensure comprehensive and accurate information gathering. In the first stage, we extracted socio-economic and clinical data from patients´ medical records, including age, gender, occupation, medical comorbidities, risk factors, details on the acute phase of infection, initial symptom presentation, and disease management during the acute phase. In the second stage, we collected data on persistent symptoms suggestive of LC through structured telephone interviews, three months after the acute phase. These interviews were carried out by three trained medical residents using a checklist developed based on a comprehensive literature review. The checklist covered symptoms duration, intensity, and management. Each interview lasted approximately ten minutes. To ensure clarity and cultural relevance, the questionnaire was translated into Tunisian dialect and pre-tested on a sample of COVID-19 patients admitted to the emergency department in March 2021. This pre-testing helped identify potential biases and assess the validity and comprehensibility of the questions. Data were initially recorded on paper before being transferred to an electronic database, ensuring participant anonymity.

**Definitions:** COVID-19 was diagnosed based on the presence of symptoms suggestive of the disease, confirmed by either a positive RT-PCR, a positive rapid diagnostic test, or characteristic signs on a chest CT scan. Long COVID was identified according to the NICE definition, which describes it as signs and symptoms that develop during or after an infection consistent with COVID-19 and continue for more than 12 weeks, and are not explained by an alternative diagnosis [8].

**Statistical analysis:** all statistical analyses were performed using the Statistical Package for Social Sciences (SPSS) Software, version 18.0. Descriptive statistics were used to summarize participant characteristics and outcomes. Continuous variables were expressed as means with standard deviation for normally distributed data and as medians with interquartile ranges for skewed distributions. Categorical variables were presented as frequencies and percentages. To examine the association between the dependent variable (occurrence of LC) and potential risk factors, we performed both univariable and multivariable analyses. Univariable analysis was conducted using Pearson´s Chi-square test or Fisher´s exact test for categorical variables and Student´s T-test for continuous variables. A multivariable binary logistic regression model was built using the Enter method, including all candidate variables identified from the univariable analysis. Variables with a p-value < 0.20 in the univariable analysis, along with those previously reported in the literature as potential predictors of LC, were considered for inclusion in the model, regardless of their statistical significance in our data. Adjusted odds ratios (aOR) and 95% confidence intervals (CI) were reported to quantify associations. The model´s goodness of fit was assessed using the Hosmer-Lemeshow test. A two-tailed p-value inferior to 0.05 was considered statistically significant for all tests. Missing data were handled using complete case analysis.

**Ethical considerations:** all participants gave verbal informed consent after being informed of the research objectives. This study was approved by the Ethical Committee of Abderrahmen Mami Hospital (approval No. 15/2021, issued on January 7^th^, 2022). The committee is chaired by a professor of pneumonology and comprises a multidisciplinary team of experts, including specialists in pneumonology, anesthesia and intensive care, microbiology, oncology, anatomo-pathology, radiotherapy, thoracic surgery, biology, and pharmacy. Additionally, the committee includes a representative of civil society, a legal expert, and a nursing executive.

## Results

During the study period, 1271 patients were admitted to the COVID unit of the Abderrahmen Mami Hospital Emergency Department, 392 patients died during their hospitalization or after their discharge, and 425 were unreachable.

**General characteristics:** a total of 454 participants were included in this study, among them 244 (53.7%) were men and 210 (46.3%) were women. The mean age of the cohort was 58.6±13.9 years, with a range of 20 to 94 years. Nearly two-thirds of patients (289) had at least one comorbidity. Hypertension was the most frequent condition (32.8%), followed by diabetes (30%) and obesity (9.3%). Patient characteristics are summarized in Table 1.

**Table 1:** characteristics of study population (N=454), COVID unit of Abderrahmen Mami Hospital Emergency Department, April-July 2021

Variable	Number	Percentage
**Age**	58.6±13.9 years*	
**Gender**		
Men	244	53.7
Women	210	46.3
**Level of education**		
Illiterate	73	17.3
Primary	151	35.7
Secondary	135	31.9
University	64	15.1
**Profession**		
Housewife	135	31.5
Middle manager	104	24.2
Retired	100	23.3
Upper manager	40	9.3
Worker	39	9.1
Unemployed	11	2.6
**Tobacco consumption**		
Not smoker	349	79.3
Ex-smoker	66	15.0
Smoker	25	5.7
**Pack-years**	38±28.9*	
**Comorbidities**		
Hypertension	149	32.8
Diabetes	136	30.0
Obesity	42	9.3
Dyslipidemia	39	8.6
Coronary artery disease	29	6.4
Asthma	25	5.5
COPD	19	4.2
Hypothyroidism	21	4.6
Rhythm disorders	18	4.0
Allergy	10	2.2
Neoplasia	8	1.8
Heart failure	7	1.5
Kidney failure	5	1.1
Psychiatric disorders	5	1.1
Bronchial dilatation	3	0.7
Diffuse interstitial lung disease	1	0.2
Tuberculosis	1	0.2
Hemopathy	1	0.2
**COVID-19 vaccination**	133	45.5
**Time of vaccination**		
Before the infection	14	10.6
After the infection	115	87.1
Before and after	3	2.3
**Vaccine type**		
Pfizer	52	49.1
Astrazeneca	29	27.4
Jonhson	11	10.4
Sinovac	5	4.7
Astra-Pfizer	3	2.8
Jonhson-Pfizer	3	2.8
Sinopharm	2	1.9
Moderna	1	0.9
**Number of vaccine doses**		
One dose	81	61.4
Two doses	47	35.6
Three doses	4	3.0

* Mean ± standard deviation; COPD: chronic obstructive pulmonary disease

**Clinical presentation and course:** the most commonly reported symptoms during the acute phase of COVID-19 were, respectively, dyspnea (85.6%), fever (53.9%), asthenia (50.3%), and dry cough (43%). Diagnosis of SARS-CoV-2 was confirmed by RT-PCR in 48.1% of cases. Chest tomography was performed in 21.8% of cases. It showed extensive lesions between 25% and 50% in 38.7% of cases and greater than 50% in 38.7% of cases. The majority of patients (77.5%) were transferred to medical services after conditioning, while a minority (8.9%) needed intensive care unit admission. The mean oxygen saturation on admission was 85.98±7.9%, and 96.3% needed oxygen therapy. Manifestations and management of COVID-19 on admission are presented in Table 2.

**Table 2:** clinical presentation of COVID-19 in study population (N=454), COVID unit of Abderrahmen Mami Hospital Emergency Department, April-July 2021

Variable	Number	Percentage
**Diagnosis confirmed by**		
RT-PCR	216	48.1
Rapid diagnostic test	135	30.1
Chest scan	98	21.8
**Extent of the thoracic CT scan**		
0-25%	25	22.5
25-50%	43	38.7
>50%	43	38.7
**Transfer**		
Medical department	348	77.5
ICU admission	40	8.9
Discharged with home oxygen therapy	26	5.8
Discharged	35	7.8
**Oxygen saturation on admission**	85.98±7.9%*	
**Symptoms of COVID-19**		
Dyspnea	386	85.6
Fever	243	53.9
Asthenia	227	50.3
Dry cough	194	43.0
Digestive problems	109	24.2
Headache	70	15.5
Influenza-like illness	60	13.3
Arthralgia	39	8.6
Chest pain	31	6.9
Myalgia	26	5.7
Altered general condition	21	4.6
Anorexia	13	2.9
Sputum	11	2.4
Chills	8	1.8
Smell disorders	7	1.6
Dizziness	6	1.3
Taste disorders	3	0.7
Hemoptysis	3	0.7
**Acute complications**	100	24.1
**Kind of complications**		
Thrombotic complications	30	32.3
Severe hypoxia	38	40.9
Both	5	5.4
Others	20	21.5
**Oxygen therapy**		
Under oxygen therapy	422	96.3
Under oxygen concentrator	24	6.8
**Oxygen flow**	6.82±4.5 l/min*	
**Oxygen therapy methods**		
Nasal cannula	198	48.8
High-concentration mask	202	49.8
Non-invasive ventilation	6	1.5
**Curative anti-coagulation**	188	42.9
**IGSA score**	3±[2-10]**	
**CCMU**		
Grade 1	2	0.5
Grade 2	22	5.0
Grade 3	276	62.3
Grade 4	143	32.3

* Mean ± standard deviation; **median ± interquartile range; RT-PCR: real-time polymerase chain reaction; ICU: intensive care unit; CT scan: computed tomography scan; IGSA: *indice de gravité simplifié ambulatoire*; CCMU: classification *clinique des malades aux urgences*

**Figure 1:**
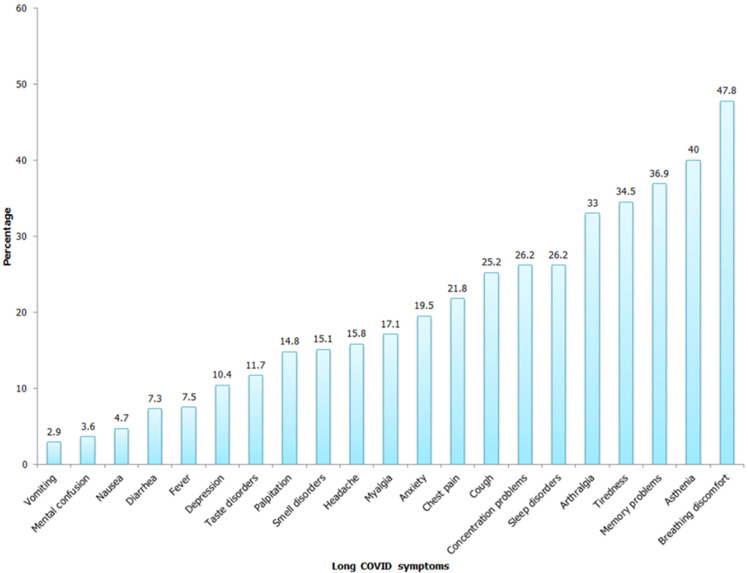
prevalence of long COVID symptoms in patients of the COVID Unit of Abderrahmen Mami hospital, April-July 2021

**Long COVID manifestations:** a total of 385 patients (84.8%) experienced LC, and one-quarter of them presented six symptoms or more. Approximately 40% of participants reported ongoing symptoms. The most common persistent symptoms were breathing discomfort (47.8%), followed by asthenia (40%), memory problems (36.9%), tiredness (34.5%), and arthralgia (33%) (Figure 1). Symptoms´ evolution was continuous among 39.6% of patients, while others reported intermittent evolution of their symptoms. Nearly two-thirds of participants (60.5%) declared experiencing fluctuating symptom intensity. Additionally, among initially symptomatic participants, less than half (43.5%) reported visiting a doctor for these persistent symptoms, 35.5% needed exploration, and 22.1% were given a treatment.

**Factors associated with long COVID:** symptoms of LC were significantly more frequent in female patients (p<0.019), participants with hypertension (p<0.033), and those with an increased number of comorbidities (p<0.027), as shown in the univariable analysis (Table 3). According to the multivariable analysis, evidence for association between women and LC was established (aOR: 1.732, 95%CI 1.002-2.995; p = 0.049) (Table 3).

**Table 3:** univariable and multivariable analysis of factors associated with long COVID, COVID unit of Abderrahmen Mami Hospital Emergency Department, April-July 2021

Variable	Univariable analysis		Multivariable analysis	
	Unadjusted ORs (95% CI)	P-value	Adjusted ORs (95% CI)	P-value
**Age**	0.996 (0.978-1.015)	0.713	1.000 (0.979-1.022)	0.995
**Women**	1.889 (1.102-3.238)	**0.019**	1.732 (1.002-2.995)	**0.049**
**Asthma**	4.521 (0.601-33.980)	0.151	4.225 (0.556-32.120)	0.164
**COPD**	0.483 (0.168-1.387)	0.187	0.544 (0.183-1.618)	0.274
**Dyslipidemia**	2.269 (0.679-7.586)	0.172	1.388 (0.355-5.425)	0.637
**High blood pressure**	1.922 (1.045- 3.535)	**0.033**	1.732 (0.933-3.218)	0.082
**Hemopathy**	-	0.152	-	1.000
**Co morbidities number**	1.265 (1.006-1.591)	**0.044**	1.138 (0.829-1.563)	0.424
**ICU admission**	1.701 (0.585-4.940)	0.324	1.026 (0.271-3.885)	0.969
**More than five symptoms in the acute phase of COVID-19**	1.946 (0.247-15.329)	1.000	0.778 (0.088-6.882)	0.821
**Acute complications**	1.600 (0.800-3.200)	0.181	1.340 (0.613-2.931)	0.463
**Curative anti-coagulation**	1.460 (0.852-2.500)	0.167	1.470 (0.852-2.535)	0.166

COPD: chronic obstructive pulmonary disease; ICU: intensive care unit; ORs: odds ratios; CI: confidence interval

## Discussion

Our study aimed to assess the prevalence and characteristics of LC in a cohort of adult patients discharged from the hospital after recovering from COVID-19 in Tunisia. Our findings revealed a high prevalence of LC (84.8%), with the most common persistent symptoms being breathing discomfort, asthenia, memory problems, tiredness, and joint pain. Additionally, female patients were found to be at a higher risk of experiencing LC compared to male patients.

Data emerging for LC prevalence are conflicting; this may be due to different follow-ups ranging from 14 days to 10 months [9-15] and populations (hospitalized, non-hospitalized, those with non-critical COVID-19, those without complications) [11-13,16,17]. Following our findings, a meta-analysis including 47,910 individuals from different parts of the world showed that 80% of previously infected patients continue to have at least one symptom two weeks after the acute phase of infection [9]. An Italian study reported a similar prevalence of persistent symptoms (87.4%) [18]. A study on hospitalized patients revealed a prevalence of 96% of one persistent symptom or more at 12 weeks of follow-up [19]. Carvalho-Schneider *et al*. found a lower prevalence of LC at two months of follow-up in patients with non-critical COVID-19 (66%) [11], Peghin *et al*. noted a lower prevalence as well (40.2%) at six months after acute infection in adult inpatients and outpatients [20]. Our prevalence may be higher due to the inclusion of a major proportion of patients requiring ICU admission (8.9%), which is associated with a greater risk of persistent symptoms at three months. This high prevalence of LC in our study population underlines the need for structured post-COVID follow-up programs.

COVID-19 patients can experience long-term complications after recovery from their illness, including a wide variety of symptoms [21]. In keeping with existing studies, we found that many patients continue to have at least one sign beyond twelve weeks following the acute infection. The five most common symptoms in the present study were breathing discomfort (47.8%), asthenia (40%), memory problems (36.9%), tiredness (34.5%), and joint pain (33%).

These findings are supported by several other studies. An extensive systematic review on LC found that fatigue and dyspnea were the most common prevalent symptoms, followed by shortness of breath, cough, joint pain, chest pain, headache, loss of smell/taste, sore throat, loss of memory and depression, and anxiety [6]. The same results were found by Carfi *et al*. [18] and Xiong Q *et al*. [22]. Another study revealed that fatigue, dyspnea, and anosmia were the predominant symptoms at 3-4 months of follow-up [17]. A living systematic review, including 32 studies that analyze LC in 10,951 people in 12 countries, reported that weakness, general malaise, fatigue, concentration impairment, and breathlessness were the most described symptoms at 12 weeks or more post-infection [16]. Another systematic review and meta-analysis concluded on similar frequent symptoms (fatigue, headache, attention disorder, hair loss, and dyspnea) [9]. Unlike our results, fatigue seemed to be the leading symptom of LC in many previous studies [20], along with respiratory manifestations [23]. Differences between studies may be due to different populations, selection criteria, instruments used to collect data, and the definition of symptoms of LC.

Several hypotheses are implicated in the development of the most frequent manifestations of LC. One of the advanced theories stated that SARS-CoV-2 enters cells via the SARS-CoV-2 receptor angiotensin-converting enzyme 2 (ACE2) [24], which explains the extension of the infection to extra-pulmonary tissues and organs [25]. Other studies suggest that there is a systemic viral persistence from the acute phase [26]. Recent findings suggest mechanisms involving the immune response to the virus. Cervia *et al*. discovered an immunoglobulin signature based on total levels of IgM and IgG3; people with lower concentrations of these two antibodies had a higher risk of developing LC [27]. The respiratory symptoms were the most reported symptoms since the SARS-CoV-2 receptors, ACE2, were highly expressed in the respiratory tract.

Respiratory outcomes in LC are common, as assessed by a study which interested in the impact of persistent lung dysfunctions among COVID-19 patients six months after hospital discharge [28]. This could be explained by viral invasion of alveolar epithelial and endothelial cells and immunological damage causing perivascular inflammation [29]. SARS-CoV-2 can also affect the cardiovascular system via different mechanisms; it causes myocardial damage and inflammation through ACE2 receptors, pericardial involvement, and vascular thrombosis [21,30]. Several studies reported cardiovascular anomalies 3 to 6 months after the COVID-19 onset [21].

Cognitive dysfunction and mental health impairments may be attributed to brain damage caused by SARS-CoV-2 directly or indirectly following a cytokine storm affecting the brain [31]. This damage to the brain may aggravate a pre-existing neurological disorder or induce a new one [32] along with immunosenescence and associated inflammaging [33].

Studies suggest that patients with severe COVID-19 disease had a higher risk of developing gastrointestinal symptoms and liver injury compared to those with non-severe disease [34]. The most common gastrointestinal sequelae in LC were loss of appetite, nausea, acid reflux, and diarrhea [35]. Another probable mechanism that could contribute to LC is gut microbiota dysbiosis that could persist after infection´s resolution [36].

A wide diversity in risk factors for LC was noted [16]. Recent studies conducted on patients recovered from COVID-19 found similar results to we did; they demonstrated that female sex was a risk factor for LC [20,37,38], while others did not conclude the same fact [15,39-41]. Available literature had identified multiple LC associated factors such as; the number of symptoms at the onset of the COVID-19 [20], ICU admission [20], older age [23], preexisting comorbidities [3], disease severity [42], hospital admission [43], oxygen supplementation at the acute phase [22], obesity [44], respiratory distress [45], long duration of the disease [45], length of hospital stay [46] and pre-existing hypertension [46].

Social factors can explain why we found that women were more prone to LC since, in general, they tend to report higher prevalence of symptoms than men due to differences in somatic and visceral perception, description, and reporting [47,48]. In addition to that, it has been demonstrated that females are frequently more affected by autoimmune diseases than males [49]. In fact, in LC, studies have shown that women presented an elevated level of the pro-inflammatory cytokine IL-6 compared to men [50]. A survey in Croatia revealed that women were at a greater risk of developing greater functional impairment in LC, but could not explain why [51].

A recent meta-analysis involving 13,340 patients in 20 studies which investigated prognostic factors for post-COVID-19 syndrome found a significant association between female sex and the occurrence of any symptom of LC which is in contrast with what evidence had shown for the acute phase where males were more at risk to develop serious COVID-19 disease, this could lead to a higher risk of mortality in men meaning that COVID-19 epidemiology does not reflect LC epidemiology [52,53]. A model using age, gender, and number of reported symptoms during the first week was established to identify patients at risk for LC, but caution is needed when generalizing this model to the general population [54]. Data are conflicting in terms of LC-associated factors; further studies are required to disclose the etiology of LC, identify more risk factors, and develop prevention strategies. In light of these data, it seems important to integrate LC assessments into routine post-COVID care, particularly for at-risk groups.

Our study has several strengths, including its focus on a previously unstudied population in Tunisia and the use of a structured approach to collect data, combining medical record extraction and telephone interviews. However, some limitations should be acknowledged. The retrospective design may introduce recall bias, particularly for self-reported symptoms during the telephone interviews. Additionally, the study was conducted in a single hospital, which may limit the generalizability of the findings to other settings or populations. Our population size is relatively small, which can hide the effect of some variables on LC. Despite these limitations, our research contributes to expanding knowledge of this emerging public health concern by providing useful data on the prevalence and features of LC in Tunisia.

## Conclusion

Our study highlights the significant prevalence of LC among COVID-19 patients in Tunisia, three months after recovery from the acute COVID-19 disease. The most frequently reported symptoms were breathing discomfort, asthenia, memory problems, tiredness, and arthralgia. The multivariable analysis identified female sex as a factor independently associated with a lack of return to a pre-illness baseline state. These findings emphasize the need for greater awareness of LC and its diverse manifestations. Further longitudinal studies are essential to fill in the gaps in the etiological factors of LC and shed light on the underlying mechanisms. Such research could help optimize patient care and reduce the burden of LC on society and healthcare systems.

### What is known about this topic

- Long COVID is a post-viral condition that could persist long after the initial infection of SARS-CoV-2;
- Long COVID affects various organ systems, resulting in a diverse range of physical symptoms;
- Risk factors for long COVID are mainly advanced age, female sex, pre-existing health conditions, and severity of the initial COVID-19 infection.

### What this study adds

- Highlights the high prevalence of long COVID among COVID-19 patients in a Tunisian hospital, three months after the acute phase;
- Highlights the most frequently reported long COVID symptoms, including breathing discomfort, asthenia, memory problems, tiredness, and arthralgia;
- Reveals an association between long COVID and female gender.
